# Insight into the effects of Omega-3 fatty acids on gut microbiota: impact of a balanced tissue Omega-6/Omega-3 ratio

**DOI:** 10.3389/fnut.2025.1575323

**Published:** 2025-05-16

**Authors:** Bo Zou, Dan Zhao, Shuang Zhou, Jing X. Kang, Bin Wang

**Affiliations:** ^1^Department of Clinical Nutrition, Shenzhen Longgang Central Hospital, Shenzhen, China; ^2^College of Food Science and Engineering, Laboratory of Functional Chemistry and Nutrition of Food, Northwest A&F University, Yangling, Shaanxi, China; ^3^Laboratory for Lipid Medicine and Technology, Department of Medicine, Massachusetts General Hospital and Harvard Medical School, Boston, MA, United States; ^4^Omega-3 and Global Health Institute, Boston, MA, United States

**Keywords:** Omega-3 fatty acids, Omega-6/Omega-3 ratio, gut microbiota, chronic low-grade systemic inflammation, experimental standardization

## Abstract

Emerging evidence suggests that Omega-3 polyunsaturated fatty acids (PUFAs) are essential structural and functional nutrients that significantly influence the composition and function of the gut microbiota, a key mediator of host health. Although evidence suggests that increasing tissue Omega-3 levels through dietary intervention may optimize gut microbiota–host interaction through modulation of composition, metabolite production, and the intestinal mucus barrier, some studies have reported inconsistent findings regarding these protective effects. Studies indicate that a high Omega-6/Omega-3 ratio appears to attenuate the beneficial effects of Omega-3 supplementation on microbial diversity and abundance, while a balanced ratio fosters a more favorable microbiome profile. This review comprehensively highlights the potential effects of differential endogenous Omega-6/Omega-3 ratios on the gut microbiota-modulating capacity of Omega-3 PUFAs, which should be incorporated as a mandatory monitoring indicator in future clinical investigations. These insights provide a new direction for further optimizing the clinical application of Omega-3 PUFAs in chronic disease prevention and treatment.

## 1 Introduction

The human gut harbors the largest and most diverse microbial community within the body and simultaneously represents its largest immune organ. Given its crucial role in maintaining host health, the gut microbiota is now a principal area of investigation in life science. Factors including age (1), gender (2), diet (3), and drug use (4) collectively influence the unique gut microbiota composition of each individual. Among these factors, diet exerts the most significant influence, supplying essential nutrients and driving dynamic changes in the gut microbiota (5). A large prospective cohort study concluded that diet has a greater influence on the gut microbiota than genetics (6). Therefore, research into the effects and mechanisms of specific dietary patterns and nutrients may provide new insights into gut microbiota modulation and chronic disease control.

Omega-3 polyunsaturated fatty acids (PUFAs) are a series of essential fatty acids, mostly found in plant seeds, algae, and marine animals, which have been associated with numerous health issues and thus garnered extensive research attention (7). Notably, the global population commonly suffers from insufficient Omega-3 PUFAs intake and body stores due to the limited dietary sources in the modern diet (8). In recent years, research linking Omega-3 deficiency to gut microbiota has been steadily increasing (9). Omega-3 PUFAs can shape microbial communities within the gastrointestinal tract, potentially enhancing microbial diversity and promoting the growth of beneficial species. These shifts in gut microbial composition are hypothesized to contribute to various health outcomes, including immune regulation, metabolic homeostasis, and mental well-being (10–13). However, some studies have yielded inconsistent results, failing to observe these significant effects, raising questions about the efficacy of Omega-3 PUFAs in modulating the gut microbiota (14, 15). Research suggests the Omega-6/Omega-3 fatty acid ratio is a critical determinant in this modulation. A high tissue omega-6/omega-3 PUFA ratio may increase proportions of lipopolysaccharide (LPS)-producing or pro-inflammatory bacteria, whereas a low ratio may promote LPS-suppressing or anti-inflammatory bacteria (16). It is postulated that a balanced Omega-6/Omega-3 ratio may influence the capacity of Omega-3 PUFAs to support a healthy gut microbial environment.

This review will delve into the effects of Omega-3 PUFAs on the gut microbiota, focusing on how variations in the Omega-6/Omega-3 ratio may contribute to the inconsistencies observed in previous studies. By examining the factors influencing Omega-3 efficacy, we aim to provide a scientific foundation for recommendations aimed at standardizing Omega-3 research and applications. Establishing standardized guidelines could enhance the reproducibility of findings and optimize the therapeutic potential of Omega-3 PUFAs in clinical settings.

## 2 Omega-3 PUFAs as prebiotics under an updated concept

**Figure 1:**
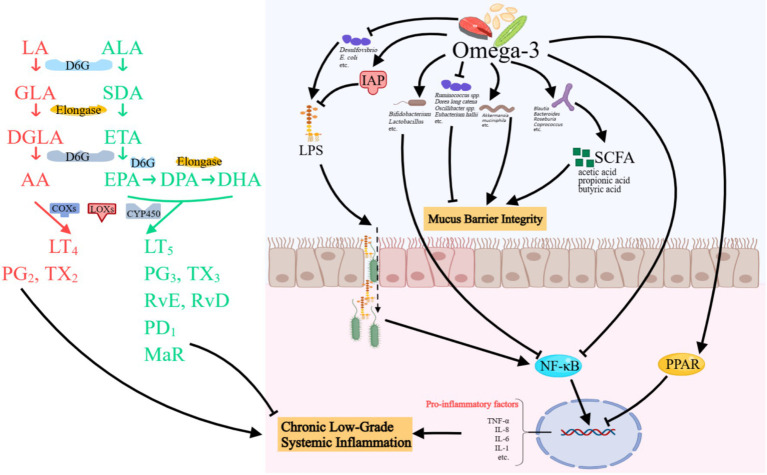
Possible mechanisms of Omega-3 PUFAs in gut microbiota modulation. Omega-3 PUFAs ameliorate intestinal inflammation by enriching beneficial bacteria (e.g., *Bifidobacterium* and *Akkermansia*). In addition, they enhance SCFA production, activate anti-inflammatory pathways, and inhibit NF-κB signaling. Omega-3 also detoxifies LPS via IAP and reduces LPS-producing bacteria. Concurrently, they strengthen mucus barrier integrity by modulating mucolytic bacteria and endothelial junctions, while specialized pro-resolving mediators resolve inflammation and promote tissue repair. LA, linoleic acid; GLA, *γ*-linoleic acid; DGLA, Dihomo γ-linolenic acid; AA, Arachidonic acid; ALA, *α*-linoleic acid; SDA, Stearidonic acid; ETA, Eicosatetraenoic acid; EPA, Eicosapentaenoic acid; DPA, Docosapentaenoic acid; DHA, Docosahexaenoic acid; COXs, cyclooxygenase; LOXs, lipoxygenase; CY, cytochrome; LT, leukotrienes; PG, prostaglandin; TX, thromboxane; Rv, resolvin; MaR, maresins; IAP, intestinal alkaline phosphatase; LPS, lipopolysaccharides; SCFA, short-chain fatty acids; NF, nuclear factor; TNF-α, tumor necrosis factor-α; IL, interleukin. Created with MedPeer.cn.

With advances in understanding nutrient–microbiome–host interactions, the concept of prebiotics has been revised to encompass substances that induce specific changes in the composition and/or activity of the gut microbiota, thereby conferring health benefits to the host (17). Therefore, although not a selectively fermented ingredient, Omega-3 PUFAs are increasingly regarded as a prebiotics under this updated concept (Figure 1).

### 2.1 Mechanisms

#### 2.1.1 Modulation of the gut inflammatory microenvironment

*Bifidobacterium* and *Lactobacillus*, two of the earliest and most intensively studied gut microbes, can reduce gut inflammation by stabilizing inhibitor of nuclear factor-κB (IκB) and suppressing the activation of nuclear factor (NF)-κB signaling pathway (18–20), which consequently leads to downregulated expression of tumor necrosis factor (TNF)-*α* and Interleukin (IL)-8 along with upregulated expression of IL-10. Omega-3 PUFAs, particularly EPA and DHA, significantly increase the abundance of *Bifidobacterium* and *Lactobacillus,* and reduce pro-inflammatory bacteria such as *Deferribacteraceae*, as demonstrated in both human cohorts and animal models of intestinal inflammation. This may occur by serving as substrates for bacterial membrane phospholipid synthesis, thereby improving their colonization resistance against pathogens (21, 22). In addition, Omega-3 PUFAs can increase the abundance of short-chain fatty acids (SCFA)-producing beneficial bacteria, leading to increased SCFA production. This enhances the function of regulatory T cells, reduces oxidative stress, and modulates the intestinal inflammatory environment (23).

LPS, derived from the outer membrane of Gram-negative bacteria, promotes inflammation and metabolic dysfunction by increasing Toll-like receptor (TLR) 4 activation (24) and activating the NF-κB signaling pathway, leading to increased levels of pro-inflammatory cytokines, including IL-1β, TNF-*α*, and IL-6 (25). Omega-3 PUFAs modulate intestinal alkaline phosphatase (IAP), an enzyme that dephosphorylates LPS, thereby detoxifying it (26). Furthermore, the bioactivity of IAP is influenced by cell membrane fluidity, and the incorporation of Omega-3 PUFAs into the membrane is crucial for modulating this fluidity, thus significantly altering IAP bioactivity (27). Additionally, Omega-3 PUFAs have been reported to reduce the abundance of LPS-producing bacteria such as *Desulfovibrio* (28) and *E. coli* (29).

In addition, Omega-3 can directly attenuate the expression of NF-κB-regulated pro-inflammatory genes and reduce intestinal inflammation by inhibiting the phosphorylation and degradation of IκB proteins and decreasing the translocation of NF-κB dimers into the nucleus (30). Concurrently, Omega-3 enhances the production of anti-inflammatory mediators such as IL-10 through PPARγ activation, which suppresses proinflammatory gene transcription (31). Furthermore, specialized pro-resolving mediators (SPMs), the metabolites of Omega-3, exert anti-inflammatory effects by reprogramming immune cells, reducing cytokines, and enhancing tissue repair without immunosuppression. SPMs also beneficially alter gut microbiota composition and strengthen intestinal barrier function. These actions collectively contribute to alleviating inflammation and maintaining gut health (32).

#### 2.1.2 Enhancement of mucus barrier integrity

The anti-inflammation properties also enable Omega-3 PUFAs to protect the gastrointestinal (GI) tract mucus barrier, serving as the primary line of defense in the digestive tract; the integrity of this barrier is crucial for maintaining host health. Compromise of this barrier leads to increased permeability, raising the likelihood that detrimental bacteria and metabolites like LPS enter the bloodstream, potentially causing chronic systemic low-grade inflammation and subsequent adverse health effects (33). An abundance of evidence shows that Omega-3 PUFAs exert a protective effect by decreasing localized inflammation, enhancing endothelial tight junctions (34), increasing the production of submucosal collagen (35), deepening the ileum recess and the jejunum villi (36), and altering the structure of the epithelial cell membrane (21).

Furthermore, Omega-3 PUFAs can directly influence the abundance of beneficial and detrimental microorganisms residing within the mucus barrier. SCFAs are metabolites produced by the gut microbiota through the fermentation of indigestible carbohydrates. These molecules play crucial roles in maintaining gut homeostasis, enhancing intestinal function, reducing inflammation, and modulating energy metabolism, acting partly through the inhibition of histone deacetylases and activation of G-protein-coupled receptors. SCFA-producing bacteria such as *Blautia*, *Bacteroides*, *Roseburia*, and *Coprococcus,* as well as SCFAs including iso-butyrate and isovalerate, have been reported to increase following Omega-3 intervention (37, 38).

Mucolytic bacteria are a distinct group of microbiome residing in the mucus layer that utilize mucin from the mucus barrier as an energy source (39). Pathogenic mucolytic bacteria can degrade the mucin layer and alter its viscoelasticity, potentially by modifying mucus pH. Subsequently, the protective mucus layer can be penetrated, enabling these bacteria to colonize the gut epithelial layer and eventually cause infection (40). Conversely, commensal mucolytic bacteria can compete with pathogens for attachment sites (41), and some beneficial microbiota can promote mucus growth and increase layer thickness (42–44). Following Omega-3 treatment, the relative abundances of potentially pathogenic bacteria, including *Ruminococcus* spp., *Dorea long catena*, *Oscillibacter* spp., and *Eubacterium hallii*, were significantly decreased. In contrast, the commensal mucolytic bacterium *Akkermansia muciniphila* showed the opposite trend, with its relative abundance increasing (45).

### 2.2 Evidence from animal studies

In C57BL/6 mice, a high-beef diet increased the abundance of potentially pathogenic bacteria, including Escherichia-Shigella, Mucispirillum, Helicobacter, and Desulfovibrio, but subsequent Omega-3 PUFAs supplementation reduced these levels. This suggests Omega-3 PUFAs can modulate the gut microbiome and microbial metabolic pathways altered by dietary factors such as high beef intake (46). Caesar et al. (47) reported that mice fed with fish oil exhibited higher gut *Lactobacillus* levels compared to those receiving a lard-based diet. Similarly, another animal study found that an Omega-3-enriched diet significantly increased Lactobacillus abundance at the genus level and enhanced cognitive function in mice. In contrast, mice consuming an Omega-3-deficient diet displayed impairments in communicative and social interactions, alongside increased depressive-like behaviors (48). Additionally, increasing n-3 PUFAs content in the diets of transition cows altered rumen bacterial composition, reduced prepartum inflammation, and improved postpartum milk protein content (49). Another study investigating the effects of EPA on liver fibrosis in rats found that EPA attenuated liver fibrosis and improved liver function by increasing SCFA-producing gut microbiota, such as *Blautia argi* and *Romboutsia ilealis* (50). In 2015, our research group demonstrated that altering the tissue Omega-6/Omega-3 ratio leads to correlated changes in the gut microbiome and fecal metabolites. This alteration included an elevated *Enterobacteriaceae* to *Bifidobacterium* ratio, increased serum LPS, lipopolysaccharide-binding protein (LBP), and trimethylamine oxide (TMAO) levels, and elevated inflammatory markers such as TNF-a, IL-6, and C-reactive protein (CRP) (51). Furthermore, Whiting et al. (35) found that mice with higher tissue Omega-3 levels were protected from chemically induced colitis, exhibited reduced levels of inflammatory markers like TNF-*α*, and showed higher ZO-1 expression, indicating improved tight junction and barrier function compared to the control mice.

The abovementioned findings offer valuable insights into the role and mechanisms of Omega-3 PUFA-mediated microbiota modulation. However, due to the physiological differences in metabolism, immunity, and host–microbe interactions between animals and humans, as well as the typically larger intervention doses used in animal experiments, the regulatory effects of Omega-3 on the gut microbiota in humans and its clinical application value still need to be confirmed by more human studies.

### 2.3 Evidence from human studies

Wang et al. (52) reported that the consumption of Omega-3 correlated with an 11–55% reduction in the risk of developing colorectal adenomas, and this relationship was influenced by the evenness of the gut microbiota. A cross-sectional study analyzed data from 876 middle-aged and elderly female twins and found that both total Omega-3 and DHA serum levels were significantly correlated with microbiome alpha diversity (Shannon index) based on 16S rRNA gene sequencing. Among those, the strongest correlations were observed with operational taxonomic units (OTUs) from the *Lachnospiraceae* family (53). Another study highlights the promotion of butyrate-producing bacteria, including *Clostridiaceae*, *Sutterellaceae*, and *Akkermansiaceae*, by Omega-3 PUFAs in healthy adults (54).

Nevertheless, results from some human intervention studies investigating the effects of Omega-3 PUFAs on the gut microbiota have shown inconsistency (54), a phenomenon that appears more common in human studies. Storm-Larsen et al. found that intervention with Omega-3 PUFAs had a negligible impact on the microbiota composition of individuals with familial hypercholesterolemia. Furthermore, the positive effects observed on blood lipids following Omega-3 PUFAs intervention were not associated with baseline gut microbiota composition or microbial changes during treatment (14). A thorough investigation into the reasons behind such disparate research outcomes is crucial for objectively evaluating the application value of Omega-3 PUFAs in gut microbiota regulation and for further standardizing research protocols and application methods.

## 3 Uncorrected Omega-6/Omega-3 ratio may be an important factor affecting the results of Omega-3 experiments

### 3.1 Opposing effects of Omega-6 and Omega-3 PUFAs on gut microbiota

Omega-6 PUFAs represent another crucial category of fatty acids, primarily including linoleic acid (LA; 18:2 Omega-6) and arachidonic acid (AA; 20:4 Omega-6). Studies specifically dedicated to the effects of Omega-6 PUFAs on gut microbiota are limited; oils rich in Omega-6 PUFAs are usually used as control or placebo treatments in Omega-3 intervention studies. From these studies, it can be found that Omega-6 and Omega-3 exert opposing effects on the regulation of gut microbiota. Ghosh et al. (28), using a *Citrobacter rodentium*-induced colitis model in mice, found that an Omega-3 intervention group showed a significant increase in fecal *Lactobacillus* and *Bifidobacterium* levels, along with reduced gut inflammation, compared to an Omega-6 group. In another study, compared with soybean oil (Omega-6 rich oil), the tuna oil (Omega-3 rich oil) group induced changes in the relative abundances of the genera *Turicibacter* and *Akkermansia*. Meanwhile, serotonin and serotonin metabolite levels in the amygdala were increased in the tuna oil group, suggesting potential modulation of the intestinal microbiota, immune system, and brain development/behavior (55).

### 3.2 Competitive interactions between Omega-6 and Omega-3 PUFAs

In addition to exerting direct opposing effects on the gut microbiota, Omega-6 can also indirectly inhibit the beneficial effects of Omega-3 through metabolic competition. Both Omega-6 and Omega-3 PUFAs are competitively incorporated into the phospholipid bilayer of cell membranes, thereby inducing distinct alterations in membrane stability, fluidity, and permeability (56). Furthermore, Omega-6 and Omega-3 PUFAs share the same set of metabolic enzymes, while exerting different pro-inflammatory biological effects through competitive binding with specific enzyme classes (57, 58). Therefore, excessive levels of Omega-6 in the body can directly affect the metabolism of Omega-3, thereby affecting its health effects.

Due to the mutually antagonistic and synergistic balance between Omega-3 and Omega-6 PUFAs in regulating immune responses, inflammation, coagulation, and other vital processes, the concept of the Omega-6/Omega-3 ratio has been proposed. This concept emphasizes the importance of maintaining a balance between these two classes of fatty acids in both dietary intake and tissue levels to synergistically achieve comprehensive health benefits. Unfortunately, over the past century, significant changes in dietary patterns have led to a notable alteration in the typical dietary Omega-6 to Omega-3 intake ratio, increasing from approximately 1:1 historically to over 15:1 in many modern Western diets (57, 59). In current dietary structures, cooking oils rich in Omega-6 PUFAs dominate in most households, and livestock and poultry products often come from animals fed grains rich in Omega-6 PUFAs, such as corn and soybeans (8). Conversely, significant sources of Omega-3 PUFAs, particularly EPA and DHA, are limited to foods like fatty fish, seaweed, and certain seeds/oils like flaxseed and perilla oil.

### 3.3 The Omega-6/Omega-3 ratio was overlooked in Omega-3 studies

Based on the preceding discussion, it is evident that both dietary intake and the tissue Omega-6/Omega-3 ratio can directly influence the outcomes of Omega-3 interventions. However, in previous research on Omega-3 PUFAs, particularly concerning gut microbiota, these factors have often been overlooked. We intended to summarize or calculate the Omega-6/Omega-3 ratio in the human studies related to Omega-3 and gut microbiota (Table 1); only one study monitored different forms of Omega-6/Omega-3 ratio after intervention and dropped to 2.8 (60). Although we cannot definitively determine whether a balanced Omega-6/Omega-3 ratio was achieved at the endpoint in other studies, considering the widespread excess of dietary Omega-6 PUFAs and insufficient Omega-3 PUFAs intake in modern diets, we speculate that due to variations in intervention doses, types of Omega-3 PUFAs used (which directly affect absorption and utilization rates), intervention durations, and differences in baseline Omega-6 intake and tissue levels among participants across studies, if an excessive Omega-6/Omega-3 ratio is not corrected during the intervention, this imbalance will likely influence the observed effects of the Omega-3 PUFAs supplementation. This phenomenon likely extends to research on the prevention and treatment of other chronic diseases with Omega-3 PUFAs. In past Omega-3 PUFAs research applications, the failure to differentiate between specific types of Omega-3 PUFAs, disregard for differences in structural forms affecting absorption, and formulation of protocols without considering baseline Omega-6 levels or the Omega-6/Omega-3 ratio may inevitably lead to biased intervention results under certain conditions. This, in turn, can contribute to inconsistency in determining the true intervention effects of Omega-3 PUFAs.

**Table 1:** Omega-3 intervention and gut microbiota in human studies.

Study (year)	Population	Dose^1^, duration	Omega-6/ Omega-3^2^	Main outcome	Ref
Lu (2024)	Patients with T2D and hypertriglyceridemia	1.86 g EPA + 1.5 g DHA + 0.24 g other Omega-3, 12w	/	Fish oil had minor effects on gut microbiota; serum TG and low-unsaturated TG species significantly decreased.	(15)
Balfegó (2016)	Drug-naïve T2DM	T2DM diet and 100 g sardines 5d/w	2.8	*Firmicutes/Bacteroidetes* ratio decreased; *Bacteroides-Prevotella* increased; plasma adiponectin increased; plasma insulin and insulin resistance decreased; no significant differences in glycemic control with the control group (T2DM diet).	(60)
Storm-Larsen (2022)	Heterozygote FH, using statins>12 m	4 g Omega-3 (0.92 g EPA, 0.76 g DHA), 3 m	/	Negligible impact on composition; positive effects on blood lipids were not associated with gut microbiota change.	(14)
Lim (2022)	LDL-C 3.06 ~ 4.51 mmol/L, aged 50–70, BMI ≤ 27.5	30 g dietary oil, 8w	/	The highest Omega-3 concentration is associated with faster and more robust responses of the gut microbiota; Omega-3 may decrease TG, apo-B, and the TC/HDL-C ratio by modulating the Clostridium genus.	(73)
Quin (2020)	Infants	Self-administered, 6 m	/	*Bifidobacterium* and *Lactobacillus* increased with higher breastmilk EPA, and DHA decreased microbiota richness in infants; no differences in sickness incidence in toddlers.	(74)
Vija (2020)	BMI 20 ~ 39.9, low fiber intake	0.5 g Omega-3 (0.165 g EPA, 0.11 g DHA), 6w	/	*Coprococcus* and *Bacteroides* increased*; Collinsella* significantly decreased; iso-butyrate significantly increased.	(37)
Awoyemia (2019)	Men aged 65 ~ 75, high CVD-risk	2.4 g Omega-3 (0.84 g EPA, 0.48 g DHA), 36 m	/	No significant changes in LBP or sCD14.	(75)
Djuric (2019)	Healthy adults	Personalized dose (2 ~ 10 g), 12w	/	*Yue* and *Clayton* community dissimilarity index increased.	(76)
Watson (2017)	Healthy volunteers aged≥50 years	4 g EPA + DHA, 16w	/	No significant changes in diversity or composition; *Bifidobacterium*, *Roseburia* and *Lactobacillus* abundance increased.	(54)
Younge (2017)	Premature infants with EN	Omega-6/3 ratio 3.75 ~ 5.1, 10w max	/	Lower *Enterobacteriaceae, Streptococcus*, and *Clostridium* abundance; greater diversity; enrichment of amino sugar and nucleotide sugar, purine and pyrimidine metabolism.	(77)
Noriega (2016)	Case report	0.6 g Omega-3, 14d	/	Diversity decreased, butyrate-producing bacteria increased; *Faecalibacterium* and *Akkermansia* decreased.	(78)
Rajkumar (2014)	Healthy adults and BMI > 25	0.18 g EPA + 0.12 g DHA, 6w	/	No effect on gut microbiota; insulin sensitivity significantly increased and hsCRP significantly decreased.	(79)
Andersen (2011)	9 ~ 18 m-old infants	1.6 g EPA + DHA, 18w	/	Fish oil supplementation affected changes in relative abundance among infants who had stopped breastfeeding before 9 months.	(80)
Nielsen (2007)	10 m-old infants	5 g fish oil, 3 m	/	Cluster analysis of DGGE gels in the fish oil group has a decisive influence on the microbiota composition.	(81)

Ref, reference; T2DM, Type 2 diabetes; d, day; w, week; FH, familial hypercholesterolemia; EPA, eicosapentaenoic acid; DHA, docosahexaenoic acid; m, month; LDL-C, low-density lipoprotein-cholesterol; TG, triglyceride; apo, apolipoprotein; TC, total cholesterol; HDL-C, high-density lipoprotein-cholesterol; BMI, body mass index; CVD, cardiovascular disease; LBP, lipopolysaccharide-binding protein; sCD14, soluble forms of CD14; EN, enteral nutrition; hsCRP, high-sensitivity C-reactive protein; DGGE, denaturing gradient gel electrophoresis.

1 per day.

2 after intervention.

## 4 Considerations for optimizing Omega-3 PUFAs research and application

### 4.1 Intervention designs for Omega-3 PUFAs should follow a dose–response relationship

In previous explorations of dose–response relationships, meta-analyses suggest that the optimal intake of EPA + DHA for lowering blood pressure may range between 2 and 3 g per day, with potential additional benefits for high-risk cardiovascular populations observed at intakes higher than 3 grams per day (61). Another review of 22 randomized controlled trials involving 1,155 cancer patients found that during chemotherapy, significant weight gain was observed in patients consuming more than 2 grams of Omega-3 supplements per day (only one study did not contain EPA and/or DHA), while no significant effects were observed at intakes below 2 g (62), suggesting that intervention dosages for diseases may need to be higher than the recommended dietary intakes and may vary by disease type.

To standardize and promote daily Omega-3 PUFAs application, the Chinese Nutrition Society’s 2023 Dietary Reference Intakes (DRIs) recommend an acceptable macronutrient distribution range (AMDR) for Omega-3 consumption of 0.5–2.0% of total daily energy intake for children, adolescents, and adults. This range corresponds to approximately 1–4 g per day (assuming an 1800 kCal/d diet), with EPA + DHA comprising 0.25–2 g (63). Notably, the AMDR primarily targets the prevention of nutrient deficiencies and reduction of chronic disease risk in the general population and may not be directly applicable to special populations or specific disease conditions. Therefore, when formulating Omega-3 PUFAs interventions for chronic diseases, such as cardiovascular diseases or cancer, dosages should be determined considering both the correction of potential deficiencies and active therapeutic goals, guided by principles of safety, efficacy, and evidence-based medicine. While current evidence suggests potential benefits of Omega-3 PUFAs interventions for gut microbiota modulation, the effective dosage for microbiota-targeted health promotion remains to be further validated in chronic disease control. Consequently, although existing recommendations like the DRIs offer guidance for healthy individuals, future research should prioritize dose–response studies across diverse populations to establish evidence-based thresholds for various health outcomes.

### 4.2 The Omega-6/Omega-3 ratio as a critical factor in evaluating Omega-3 interventions

Considering the prevalent overabundance of Omega-6, the resulting elevated Omega-6/Omega-3 ratio in modern diets, and significant inter-individual differences in baseline Omega-6 and Omega-3 levels, a standardized intervention protocol may result in varying final Omega-6/Omega-3 ratios among participants. Furthermore, commonly used intervention dosages and durations may not guarantee significant alterations in the Omega-6/Omega-3 ratio by the endpoint of the study, potentially leading to divergent clinical outcomes. For instance, in a study on major depressive disorder (MDD), authors assessed the somatic symptoms using the Neurotoxicity Rating Scale (NRS) and found no significant difference between the intervention (3 g Omega-3; 2 g EPA and 1 g DHA) and placebo groups after 12 weeks (64). In contrast, the opposite results were obtained in another trial (65) using a similar duration and slightly lower dose (2.4 g Omega-3: 1 g EPA, 0.75 g DHA), where a significant decrease in Children’s Depression Inventory (CDI) scores was found. Subsequent analysis revealed no significant change in the erythrocyte membrane Omega-6/Omega-3 ratio between groups in the first study, whereas a significant decrease occurred in the second study.

Therefore, the authors propose that investigating the health effects of Omega-3 PUFAs, particularly in relation to the gut microbiota, should measure tissue levels of Omega-3 and Omega-6 PUFAs before and after intervention and calculate the Omega-6/Omega-3 ratio. Clinical research on Omega-3 PUFAs or personalized management teams can utilize efficient methods like gas chromatography–mass spectrometry (GC–MS) to detect baseline levels and formulate tailored intervention strategies. During the intervention, restoring and maintaining a balanced Omega-6/Omega-3 ratio should be a primary quality control indicator. Additionally, regular monitoring of PUFA levels and the Omega-6/Omega-3 ratio allows for objective evaluation of participants’ compliance. Adjusting intervention strategies based on changes in the Omega-6/Omega-3 ratio helps ensure the effectiveness and safety of the Omega-3 PUFAs intervention (e.g., avoiding potential issues of Omega-3 excess or an overly inverted Omega-6/Omega-3 ratio). Moreover, considering that Omega-3 and Omega-6 PUFAs exist in bound and free forms and undergo dynamic synthesis, degradation, and metabolism, simultaneous detection of PUFA levels and their metabolites in different tissues and forms could provide richer information for understanding the health value and mechanisms of action of Omega-3 PUFAs.

### 4.3 Validation of Omega-3 efficacy using the fat-1 mouse model

Validating the relationship between dietary nutrients and health promotion is inherently complex due to variations in dietary backgrounds, challenges in ensuring participant compliance, and intricate interactions among nutrients. Furthermore, within academia and research, there has sometimes been confusion between nutrients and their food sources, such as the relationship between Omega-3 PUFAs and fish oil. Therefore, effectively controlling for confounding factors and conducting “head-to-head” studies targeting specific nutritional science questions at the organism level is crucial for objectively assessing the health value of target nutrients.

Our group pioneered the international establishment of the fat-1 transgenic animal model. This model involves transferring the fat-1 gene from the nematode *C. elegans* (which encodes an enzyme capable of converting Omega-6 PUFAs into Omega-3 PUFAs) into mice. This enables the generation of fat-1 mice (exhibiting an endogenous tissue Omega-6/Omega-3 ratio of approximately 1:1) and wild-type mice (with a ratio of approximately 20–50:1) within the same strain. Under the same dietary interventions, these two types of mice exhibit different metabolic characteristics and health statuses (66). Hybridizing the fat-1 model with various disease models allows for assessment of the significance of maintaining a balanced Omega-6/Omega-3 ratio in the prevention and treatment of chronic diseases. To date, the fat-1 model has been utilized in over 80 laboratories worldwide across more than 50 types of disease models, resulting in over 200 scientific publications (67, 68). The scientific evidence obtained from these studies provides robust support for elucidating the mechanisms and scientific value of Omega-3 PUFAs, thereby facilitating the standardization and translation of their application.

Several research teams have applied the fat-1 model to evaluate the impact of the Omega-6/Omega-3 ratio on gut microbiota and health more scientifically and objectively. These experimental results confirm the positive effects of a reduced Omega-6/Omega-3 ratio on gut microbiota and health. Célia *et al*. fed male fat-1 and wild-type (WT) mice either a high-fat/high-sucrose (HFHS) or a control diet. The HFHS-fed-fat-1 mice maintained normal mucus barrier function and significantly lower endotoxemia, while the HFHS-fed WT mice exhibited higher gut permeability and plasma LPS concentrations. After 18 weeks, the HFHS-fed WT mice developed obesity, glucose intolerance, and hepatic steatosis. Notably, fecal microbiota transplantation from fat-1 mice to WT mice reversed weight gain and normalized glucose tolerance along with intestinal permeability. The team concluded that Omega-3-mediated alteration of gut microbiota prevented metabolic syndrome in fat-1 mice (69). Other studies using this model showed that Omega-3 PUFAs enrichment or treatment with resolvins attenuated disruptions in intestinal homeostasis caused by ethanol consumption and systemic inflammation, with a concomitant reduction in liver injury (70).

In 2022, we used the fat-1 model to test the hypothesis that a decreased Omega-6/Omega-3 ratio could significantly reduce Irinotecan (CPT-11)-induced Gastrointestinal toxicity (GIT), including weight loss, bloody diarrhea, gut pathological changes, and mortality. The balanced Omega-6/Omega-3 ratio altered the gut microbiome profile, reduced the abundance of beta-glucuronidase (GUSB)-producing bacteria and GUSB activity, potentially decreasing the conversion of inactive SN-38G to toxic SN-38. These alterations, along with other potential gut-microbiota-independent mechanisms, reduced mucosal injuries, mucosal inflammation, goblet cell dysfunction, impairment of the gut barrier, and systemic endotoxemia, ultimately preventing CPT − 11-induced gut toxicities (71).

## 5 Conclusion and perspectives

The beneficial health effects of Omega-3 PUFAs are well-established, but the underlying mechanisms are still being elucidated and expanded upon. Research into the mechanisms by which Omega-3 PUFAs modulate the gut microbiota is still in early stages; while promising, significant further investigation is required. The effective elimination of confounding factors is the premise for evaluating the effect of dietary intervention. For the first time, we proposed that fully considering the correction of the background Omega-6/Omega-3 ratio will help to better reveal the regulatory mechanism of Omega-3 PUFAs on gut microbiota and provide a theoretical basis for the scientific health-promoting target of Omega-3 PUFAs. In future, we look forward to more research investigating Omega-3 PUFAs and the gut microbiota from the perspective of varying dietary and tissue Omega-6/Omega-3 ratios.

Omega-3 PUFAs share a reciprocal relationship with gut microbiota. While this review primarily discussed the unidirectional influence of the Omega-6/Omega-3 ratio on gut microbiota and human health, the microbiota can, in return, affect the absorption, bioavailability, and biotransformation of PUFAs (72). It has been shown that gut bacteria can release PUFA-derived metabolites that possess novel bioactivities—a process termed PUFA biotransformation. Strains such as *Butyrivibrio fibrisolvens*, *Clostridium proteoclasticum*, and *Lactobacillus Plantarum* have been implicated in such biotransformations. Thus, an imbalance in the Omega-6/Omega-3 ratio may contribute to a vicious cycle involving gut microbiota dysbiosis and potentially reduced Omega-3 PUFAs absorption or efficacy. Taken together, the restoration and maintenance of a balanced Omega-6/Omega-3 ratio should be regarded as one of the primary objectives in the design of studies and clinical applications involving Omega-3 interventions, so as to optimize the beneficial effects of Omega-3 PUFAs in gut microbiota modulation and overall health promotion.
